# A noteworthy issue: microbiome data variation depending on sampling methods in skin microecology studies in acne vulgaris patients

**DOI:** 10.3389/fimmu.2025.1566786

**Published:** 2025-06-09

**Authors:** De-Tian Xu, Qi Chen, Jia-Yi Yang, Guo-Rong Yan, Ling-Lin Zhang, Xiao-Jing Liu, Pei-Ru Wang, Jia Liu, Xiu-Li Wang

**Affiliations:** ^1^ Shanghai Skin Disease Hospital, Tongji University Medical School, Shanghai, China; ^2^ The Ice Dermalab, Shanghai, China; ^3^ Shanghai Children's Medical Center, Shanghai Jiao Tong University School of Medicine, Shanghai, China

**Keywords:** skin microecology, acne vulgaris, sampling method, microbiome, comedo extraction

## Abstract

**Introduction:**

Skin microecology significantly affects health, with the microbiome being a complex community of microorganisms. Different niche preferences of microorganisms raise concerns about the adequacy of common sampling methods like swabbing and cyanoacrylate biopsy. In this study, we aim to contribute to a more suitable sampling strategy in acne microbiome studies.

**Methods:**

This study involved ten mild to moderate acne patients. Three sampling methods were used: swab sampling (S1), modified standardized skin surface biopsy (S2), and individual comedo extraction (S3). DNA was extracted and sequenced to analyze the microbiome data.

**Results:**

There were significant differences in the bacterial and fungal microbiome data obtained by the three different sampling methods. *Staphylococcus* spp. (significantly higher in S3, *P*<0.05) and *Malassezia* spp. (higher in S3, *P*<0.05) were most affected by sampling methods. Bacterial phyla Proteobacteria (abundant in S1) and Bacteroidota (dominant in S2) also showed method-dependent variations.

**Conclusion:**

The choice of sampling method significantly impacts microbiome data, highlighting the need for accurate sampling to understand the relationship between the skin microbiome and acne. Standardizing sampling methods in future studies is essential for advancing skin microecology research.

**Clinical trial registration:**

http://www.chictr.org.cn, identifier ChiCTR-CPC-17012398.

## Highlights

- Question: In acne skin microbiome studies, are the data obtained from the samples able to represent each other? Is it necessary to distinguish samples of lesional from non-lesional follicles, and skin surface from inside follicles?
- Findings: The choice of sampling method significantly impacts microbiome data, highlighting the need for accurate sampling to understand the relationship between the skin microbiome and acne.
- Meaning: Standardizing sampling methods in future studies is essential for advancing skin microecology research.

## 1 Introduction

Skin microecology is a crucial factor that affects skin health. Hence, it has gained popularity in recent years and a considerable number of studies have been published. Skin microecology is a system involves various skin microorganisms that form a complex community in which the microorganisms may cohabitate, accrete and/or compete against each other to reach a balance. The collective term for these microorganisms is the ‘microbiome’. Throughout these studies, various sampling methods, namely swabbing, pore strip, cyanoacrylate biopsy and a modified Kligman sampling method (liquid scrubbing) were commonly utilized (1). However, due to the fact the different microorganisms may occupy their specific residential niche, these methods may be unable to distinguish the microorganisms from different niches (2).

**Figure 1:**
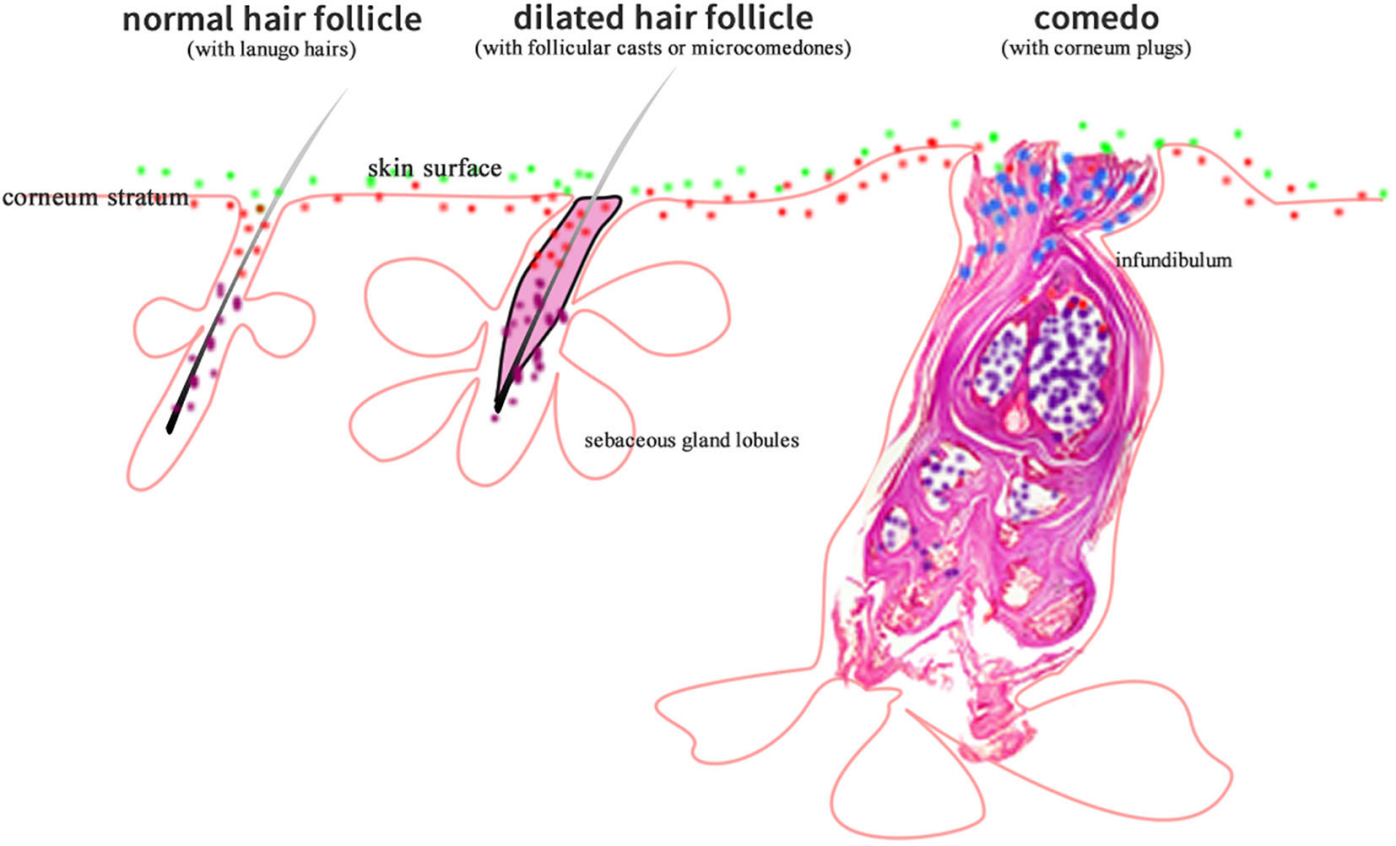
The diagram showing that aerobic (green dots), anaerobic (purple dots), microaerobic (blue dots), and amphimicrobian (red dots) microorganisms distribute in their respective skin niches.

According to preferred living conditions, skin microorganisms may distribute at skin surface, within stratum corneum, in hair follicles (either the upper/orifice or lower parts according to the nature of being aerobic or anaerobic), or in sweat gland ducts. A number of skin microorganisms have been isolated, cultured, and their nature confirmed (1), such as *Propionibacterium acnes* (*P. acnes*), *Staphylococcus epidermidis* (*S. epidermidis*), *Corynebacterium* spp., and *Malassezia* spp. etc (3). P*. acnes* is anaerobic, therefore it distributes mainly in the deep follicular part where lacks oxygen; *Corynebacterium* spp., and *Malassezia* spp. are aerobic (**Figure 1**). As a result, they live at the more superficial part of skin where oxygen is available. As for *S. epidermidis*, both aerobic and anaerobic environments are suitable for its growth.

In certain skin diseases or conditions, some microorganisms may abnormally increase or reduce in their niche, therefore causing dysbiosis, which means that the balances between different microorganisms are changed to a state that is unfriendly to health. As an example, acne vulgaris is long believed to be related to skin dysbiosis (4). Acne vulgaris primarily involves dysfunction of the pilosebaceous unit (PSU). From a histopathological view, the primary changes of this disease include over-active sebaceous gland functions in sebum production, hypercornification/proliferation of keratinocytes (or outer-root sheath cells, which originate from epidermal keratinocytes), and hypercolonization/proliferation of certain microorganisms within hair follicles (HF), thus induce the formation of acne characteristic histological structure, corneum plugs, that obstruct the follicular ducts and prevent the sheading off of all products (both from patients and microorganisms) within PSUs. At the same time, inflammation is somehow triggered (5).

The classic route to quantify the constituents of skin microbiome include a series of procedures, in brief, sampling, DNA extraction, high-throughput sequencing, and data analysis. Many standard tools and kits have been developed for the latter three procedures, however, the influence of sampling methods on analysis results were not much considered. This is especially an issue in the case of acne related microbiome studies. A series of studies in acne related microbiome were published in the past 20 years. Throughout these studies, various sampling methods, namely swabbing, pore strip, cyanoacrylate biopsy and a modified Kligman sampling method (liquid scrubbing) were utilized (6–8). However, due to the fact that different microorganisms may live in their specific residential niches as aforementioned, whether these conventional methods are able to adequately differentiate niche-specific microbiota remains a question.

We propose this question because the above-mentioned sampling methods all obtain samples indistinguishably from a certain skin area. Namely, they are not able to collect microorganisms from a certain targeted niche. In acne studies, for instance, swabbing and modified Kligman sampling method collect samples from skin surface (and may be to some extent from upper stratum corneum) but not from inside hair follicles, while pore strip and cyanoacrylate biopsy obtain samples from inside hair follicles, but not from not skin surface. The characteristics of pore strip and cyanoacrylate biopsy may be advantageous in acne studies; however, they do not differentiate samples of lesional follicles from healthy ones.

For follicle-related diseases, the microbiome data from lesional follicles may be of greater significance and of more relevance to the diseases. The microbiome within the lesional follicles may differ significantly from that of non-lesional follicles and skin surface, just as the case of ultraviolet induced fluorescence (UVF) in acne skin that we previously reported. Microorganisms can emit ultraviolet fluorescence, and various colors may indicate different microorganisms (9). Xu et al. isolated 276 corneum plugs from comedones of acne patients and observed that non-red UVF was dominant in acneic hair follicles while red UVF was dominant in non-acneic hair follicles (10). These findings once again arouse the importance of differentiating acne lesional and non-lesional hair follicles when a comparative study is carried out.

In this study, we aim to answer the following questions by comparing the microbiome data of samples obtained by different methods: In acne skin microbiome studies, are the data obtained from different sampling methods able to represent each other? Is it necessary to distinguish samples of lesional from non-lesional follicles, and skin surface from inside follicles? We believe the answers may contribute to a more suitable sampling strategy in acne microbiome studies and thus to facilitate our understandings of the relationship between skin microbiome and acne.

## 2 Methods

### 2.1 Study subjects

The study protocol was approved by the Ethics Committee of Shanghai Skin Disease Hospital (No. 2017-009) and was registered on the Chinese Clinical Trial Registry (No. ChiCTR-CPC-17012398). Ten acne patients of mild to moderate degree were recruited via the Internet and were evaluated by dermatologists. Written informed consent were obtained from each patient. The inclusion criteria were as follows: (1) Individuals over 18 years of age with acne vulgaris on their face were included; (2) Those who had read the instructions and were willing to follow the program requirements. The exclusion criteria were as follows: (1) Patients who had received oral isotretinoin, oral or topical retinoids or antibiotics, oral contraceptives, skin peeling, or had undergone local or facial surgeries or aesthetic procedures in the last 3 months; (2) Female patients who were pregnant or lactating. (3) Patients who were diagnosed of endocrine or genetic diseases that resemble acne vulgaris, such as polycystic ovarian syndrome (PCOS). (4) Rosacea, seborrheic dermatitis, perioral dermatitis and other inflammatory skin conditions that may affect the diagnosis of acne.

### 2.2 Sampling methods

The patients were instructed to avoid all color cosmetics, deodorants, sunscreens and skin creams for 24 hours prior to sampling. All patients cleaned their faces with soap gently and then were acclimated in a temperature (25°C) and humidity (50% ± 5%) controlled room for at least 30 minutes. Then their face images were documented by using a VISIA ™ system (Canfield, USA).

The skin of each patient was sampled in three ways, mainly on the cheeks and forehead, depending on the location of acne lesions. The three sampling methods were as follows:

#### 2.2.1 Swab sampling method (S1)

Samples were obtained from 4 cm^2^ areas from each subject using sterile cotton swabs. The skin area was rubbed 20 times: 10 times in one direction and 10 times perpendicular to this direction. The swabs were placed in sterile Hank’s buffered saline solution and stored immediately at -20°C until DNA extraction.

#### 2.2.2 Modified SSSB sampling method (S2)

This is a method that we modified from standardized skin surface biopsy (SSSB) in order to avoid the irritation of acrylic acid glue to eyes. Pre-experimentation involved testing gelatin concentrations (10%, 20%, 30%) to optimize follicular cast adhesion while minimizing irritation. A 20% gelatin (Sinopharm, Shanghai, China) concentration with 2% pentanediol (2%, Symrise, Germany) was selected for its balance between adhesion and safety. 0.1% carbon powder was added as pigment. Gelatin solution was stirred and dissolved at 60°C followed by high pressure sterilization at 121°C for 30 minutes. Before sampling, the solution was coated on a piece of cigarette paper of 5cm×10cm in size, then the side with the solution was applied to the skin sampling area and was peeled off after drying for 15-20 minutes to obtain follicular casts from hair follicles. We then stick the back side of the paper (the side without gelatin) flat in a sterile plastic dish, sealed and stored it at -20°C for further analysis.

In the UVC-sterilized laboratory, place the dish under a stereomicroscope. The follicular casts were carefully clamped and stripped off one by one from the gelatin film interface using sterilized precise microsurgical forceps with a swift horizontal motion. Thus, a pure intrafollicular sample without hair shafts above the skin surface was obtained. The follicular casts were then stored at -20°C until DNA extraction.

#### 2.2.3 Individual comedo extraction sampling method (S3)

Use an acne extractor to extract the corneum plugs from comedonal follicles as we previously reported (10, 11). The corneum plugs were only sampled from comedonal lesions, the primary lesion of acne. About 10 plugs were extracted from each patient. The intact plugs from each patient were sealed in a 1.5ml sterile Eppendorf vial and were stored immediately at -20°C until DNA extraction. The extraction method allows for precise access to samples within lesional hair follicles. Our previous studies (10, 11) have confirmed that careful extraction is able to obtain the whole intact comedo corneum plugs and to retain the microorganisms *in sito*.

### 2.3 DNA extraction, sequencing and analysis

Sample DNA was extracted using E. Z. N. A. ^®^ Stool DNA Kit (Omega Bio-tek, Norcross, GA, USA) according to the supplier’s instructions. The extracted DNA was barcoded with an 8 bp sequence, then was amplified in a polymerase chain reaction (PCR) amplifier (ABI GeneAmp^®^ 9700). The custom primer set 341F/907R was used to target the bacterial hypervariable regions V3-V4 of 16S rRNA gene and the primer set ITS1F/ITS2R targeting the fungal 18S-ITS. The conditions and primer sequences for each of the PCR amplifications are listed in **Table 1**.

**Table 1:** Amplification conditions of PCR in this study.

Target genes	Primers	Sequence	PCR conditions
Bactirial 16S rRNA	341F 806R	5’-CCTATYGGGRBGCASCAG-3’ 5’-GGACTACNNGGGTATCTAAT-3’	95°C for 2min, 25×(95°C 30s, 55°C 30s, 72°C 30s), 72°C for 5 min.
Fungal DNA 18S-ITS	ITS1F ITS2R	5’-CTTGGTCATTTAGAGGAAGTAA-3’ 5’-GCTGCGTTCTTCATCGATGC-3’	95°C for 2min, 29×(95°C 30s, 55°C 30s, 72°C 30s), 72°C for 5 min.

The DNA was sequenced with a next generation sequencing (NGS) high throughput system Illumina PE250, and microbiome data such as α-Diversity and β-Diversity were analyzed based on operational taxonomic units (OTU). The detailed protocol of DNA amplification, purification, sequencing, and analysis is provided in **Supplementary Material**.

### 2.4 Statistical analysis

Principal component analysis (PCA) was performed using the R package vegan. Data normalization and distance matrices were calculated prior to PCA. Visualization was done using ggplot2. Shannon-Wiener curves was generated with QIIME2 to assess sequencing depth adequacy. Venn diagrams was created using the VennDiagram package in R to illustrate OTU overlaps. α-diversity was analyzed using the Kruskal-Wallis test with Dunn’s *post-hoc* correction.

β-diversity was applied to Bray-Curtis distances. For all statistical comparisons, p-values below 0.05 were considered statistically significant.

## 3 Results

We collected and analyzed skin samples from 10 acne vulgaris patients. From each patient at the same skin area, one sample was obtained by one sampling method and three methods (S1, S2, S3) were used to obtain 30 samples in total.

### 3.1 Intrafollicular samples are effectively collected by modified SSSB method

The patient comments and our observations proved that the modified SSSB method is safe, effective, minimally invasive, and thus is friendly both to the patients and the investigators. The microscopic observation under both white and ultraviolet (peaks at 365nm) confirmed that follicular casts were effectively pulled out from inside hair follicles (**Supplementary Material S1**). This is mainly due to the partial lipophilicity of the membrane formed by gelatin (12).

Using this modified SSSB method, we successfully obtained abundant intrafollicular samples from normal follicles and microcomedones but not typical well-developed comedones.

### 3.2 Quality control, quantification of extracted DNA

**Figure 2:**
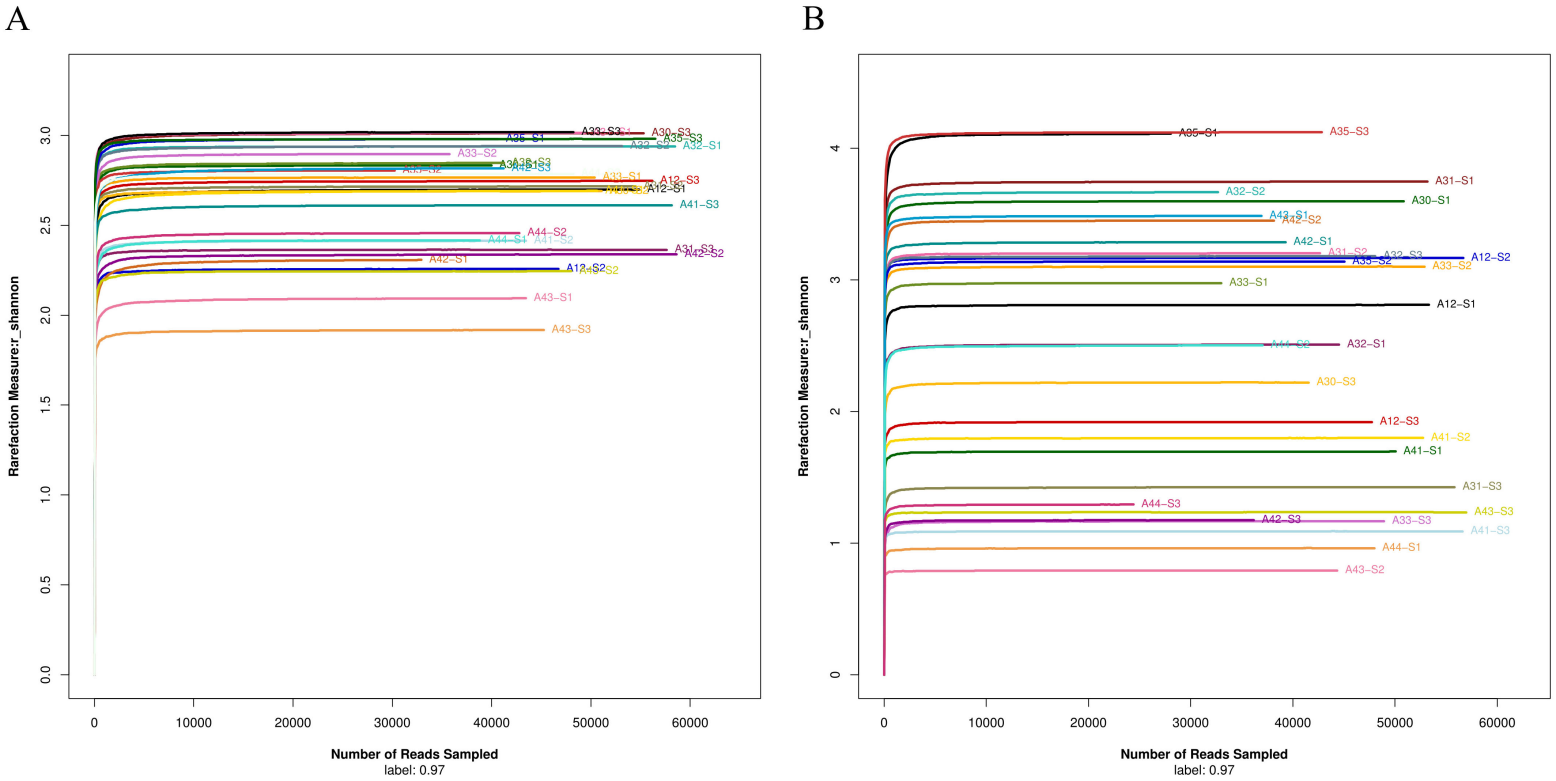
**(A)** Shannon-Wiener curve of bacterial 16S rRNA V3-V4 region sequencing data. **(B)** Shannon-Wiener curve of fungal 18S-ITS sequencing data.

After DNA extraction and amplification, sequencing, and data optimization, the DNA sequence quality was examined. The DNA amounts of all samples were enough for sequencing. The average length of 16S rRNA V3-V4 region was 416.56 bp and 18S-ITS was 263.91 bp (**Table 2**). Shannon-Wiener curve indicates that the sequencing number (30,000) in this study was sufficiently large (**Figures 2A, B**).

**Table 2:** Statistical Analysis of Optimized Bacterial and Fungal Sequencing Data.

Sample Source	Number of Samples	Number of Sequences (bp)	Base Pairs (bp)	Average Length (bp)
bacterial 16S rRNA(V3-V4)	30	1454362	605833335	416.56
fungal 18S ITS	30	1406999	371323734	263.91

### 3.3 Bacterial microbiome data analyzed from samples obtained by different methods are significantly discrepant

Bacterial diversity at phylum, family, and genus levels were analyzed from samples obtained by three different methods, namely S1, S2, and S3. The data were further compared on both an individual volunteer and group basis. The data suggest that intrafollicular bacterial microbiome data analyzed from samples obtained by different methods are significantly discrepant.

**Figure 3:**
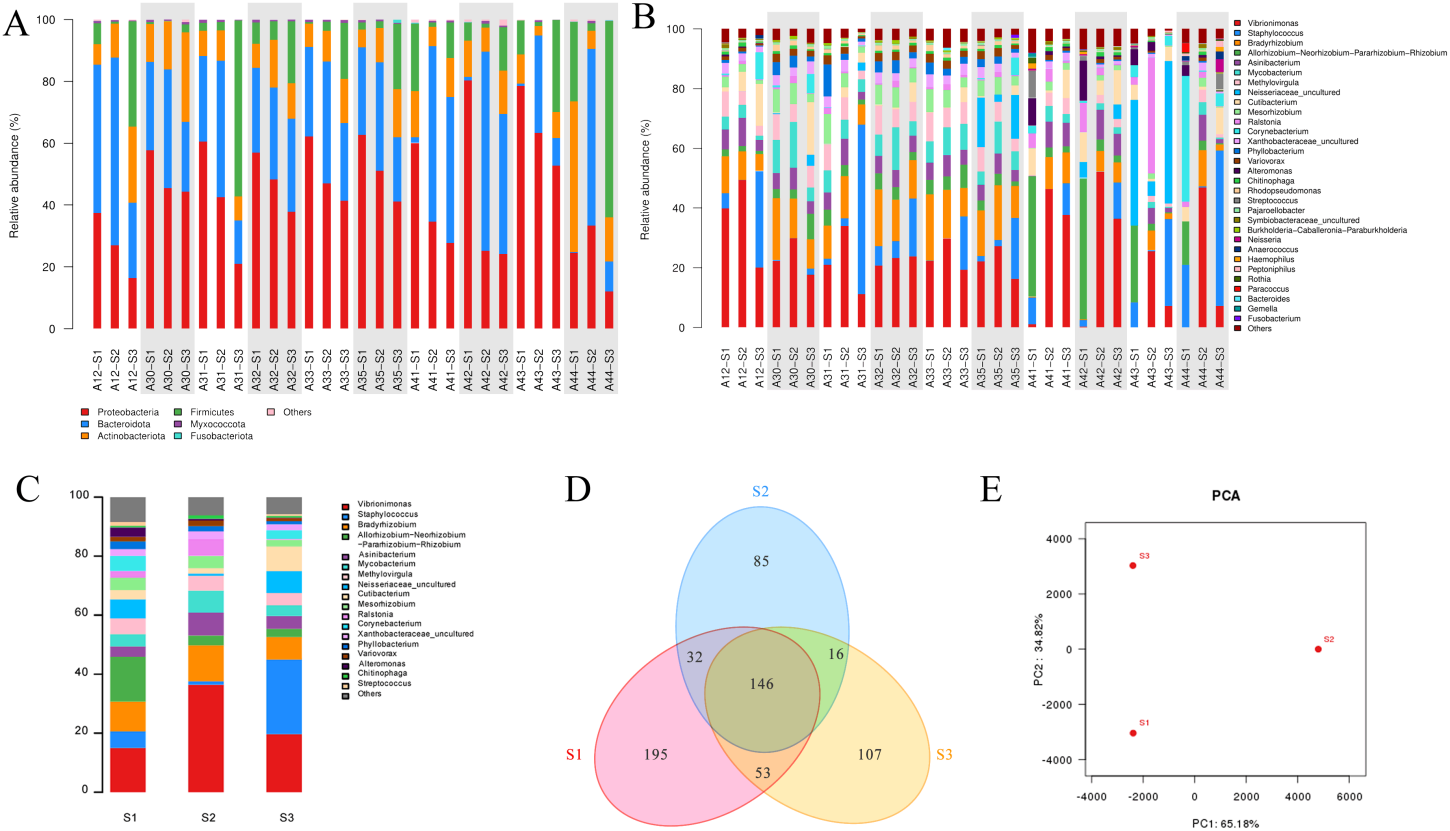
Analysis of bacterial community structure in acne patients’ skin samples obtained by three methods. **(A)** Individual analysis at phylum level. **(B)** Individual analysis at genus level. **(C)** Pooled group analysis at genus level. **(D)** Venn diagram of OTU distribution of three sample groups. **(E)** PCA analysis between S1, S2, and S3 groups. *A+number represents the volunteer’s code; S1, S2, S3 represent samples obtained by swabbing, modified SSSB method, and individual comedone extraction method, respectively.

At phylum level, when all samples are put together, the most predominant phyla are *Proteobacteria, Bacteroidota, Firmicutes*, and *Actinobacteriota*. *Proteobacteria* is found to be more abundant in skin surface samples (S1) than in follicular casts (S2) and comedonal lesions (S3). In S2 samples, *Bacteroidota* is the most abundant while *Firmicutes* the least. *Actinobacteriota* varies according to individuals (**Figure 3A**).

At genus level, the most prominent genera include *Vibrionimonas* spp.*, Staphylococcus* spp.*, and Bradyrhizobium* spp. (**Figure 3B**). *Vibrionimonas* spp shows a higher relative abundance on the skin surface (S1) and lower in follicular casts (S2). *Staphylococcuss pp.* is higher in comedonal lesions (S3) and lower in skin surface (S1) or follicular casts (S2).

β-diversity represents the biodiversity between different individuals or groups. The composition of the bacterial microbiome at the genus level is represented in **Figure 3C**, where *Vibrionimonas* spp. is higher in group S2, *Staphylococcus* spp. is significantly higher in group S3(*P*=0.000216), and *Allorhizobium* spp. is higher in group S1. A Venn diagram describes the overlap and differences in microbial species among the three groups of samples, showing that the microbial data obtained by the three groups differ from each other (**Figure 3D**). PCA shows that the microbiome data obtained from the three sampling methods differ significantly between each other (**Figure 3E**).

### 3.4 Analysis of Intrafollicular fungal microbiome

**Figure 4:**
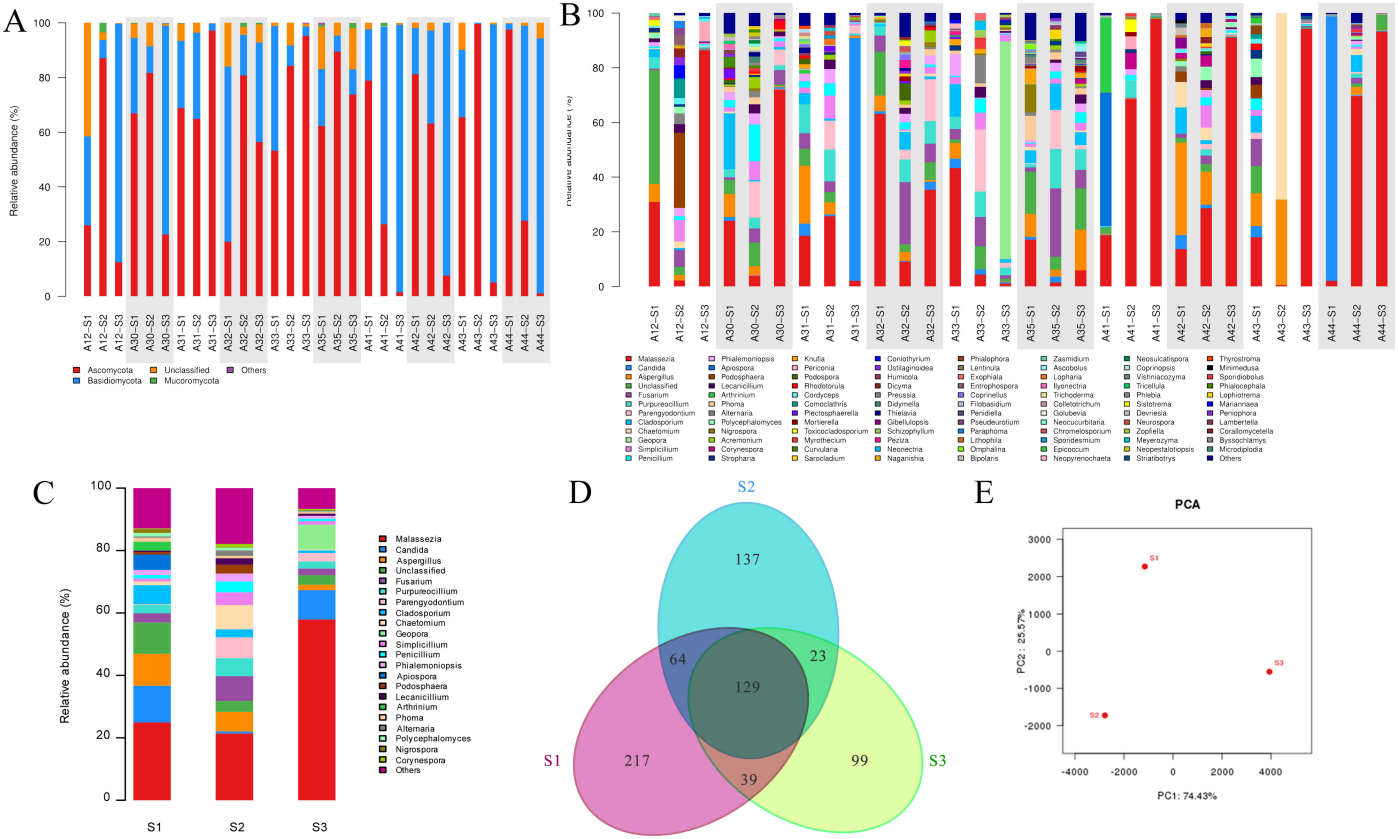
Analysis of fungal community structure in acne patients’ skin samples obtained by three methods. **(A)** Individual analysis at phylum level. **(B)** Individual analysis at genus level. **(C)** Group analysis at genus level. **(D)** Venn diagram of OTU distribution of three sampling method groups. **(E)** PCA analysis. *A+number represents the volunteer’s number; S1, S2, S3 represent samples obtained by swabbing, modified SSSB method, and individual comedone extraction method, respectively.

Taking all samples data together, the main fungi in all samples at phylum level are *Ascomycota, Basidiomycota, Chytridiomycota, Mucoromycota*, and *Olpidiomycota*, as well as a certain amount of unidentified fungi. The relative abundance of these fungi varies significantly in the same patient’s skin surface, follicular casts, and comedone corneum plugs (**Figure 4A**). Data at genus level have similar findings, among which the trends of *Malassezia* spp. and *Candida* spp. are particularly noticeable (**Figure 4B**). The relative abundance diagram of three sample groups allows for a clearer observation of these trends (**Figure 4C**).

A comparative analysis of the data obtained by the three sampling methods at the genus level shows distinct differences between the groups. *Malassezia* spp. is comparable in group S1 and S2, but is significantly higher in group S3 (*P*=0.019). *Candida* spp. is comparable in groups S1 and S3, but is significantly lower in group S2. Similarly, Venn diagram analysis and PCA analysis reveal significant differences in the fungal community data in samples obtained by three sampling methods (**Figures 4D, E**).

## 4 Discussion

In order to confirm whether sampling methods may affect the microbiome analysis results in acne skin microbiological studies, we used three different methods to collect samples from the skin of acne patients, obtaining samples from the skin surface inside hair follicles, and precisely comedonal lesional hair follicles respectively by swabbing (S1), modified standardized skin surface biopsy (S2), and individual comedone extraction (S3). High-throughput NGS was used to analyze the samples and data were compared on sampling method basis.

Significant differences in microbiome data between S1, S2, and S3 were found. As to bacterial abundance, *Vibrionimonas* spp. is higher in group S2, *Staphylococcus* spp. is significantly higher in group S3 (*P*=0.000216), and *Allorhizobium* spp. is higher in group S1. In terms of fungi, *Malassezia* spp. is comparable in group S1 and S2, but is significantly higher in group S3 (*P*=0.019). *Candida* spp. is comparable in groups S1 and S3 but significantly lower in group S2.

These findings strongly suggest that the variation of microbiome data depending on sampling methods is a noteworthy issue in skin microbiome related studies, especially when skin appendages such as hair follicles are involved.

Our results align with the views of some previous researchers, stating that different sampling methods may affect the results of microbiome analysis (13, 14). The microbiome data differences may be explained by the interactions between skin microenvironment and the microorganisms surviving in it.

The skin surface is an aerobic environment with relatively less sebum and more electrolytes and ions derived from sweat. In contrast, hair follicles contain more sebum, with the lower part being anaerobic, the upper-middle part largely oxygen-deprived, and the uppermost part aerobic, lacking sweat components. Within comedone corneum plugs, there may be more thrived microbial activity, more microbial metabolic products, and more sebum-derived products accumulation due to the obstruction by the plugs. In non-comedonal hair follicles, the flow of sebum and shedding off of cells are not obstructed, thus the accumulation of sebum, cells and microorganisms is less prominent. Therefore, the different environment conditions may profoundly shape the microbial structure and activities in various skin niches (15). Indeed, our results confirm that the microbial community compositions are significantly different in these various niches, which represent different skin microenvironmental conditions.

However, there were limitations in this study, e.g. small sample size and inclusion of only mild-to-moderate acne patients. In future research, larger cohorts, longitudinal sampling, integration of metabolomics, and standardization of S3 for clinical use are needed.

Some researchers have conducted studies on the microecology within hair follicles for other skin conditions to explore the possible relationship between the diseases and microecology. For example, Ring et al. (16) compared the differences in microecology within hair follicles between patients with pyoderma fistulas and healthy individuals, finding that certain species (*Corynebacterium., Porphyromona., Peptoniphilus.*) were more abundant in lesional hair follicles. Ho et al. (17) studied the microecology within hair follicles of patients with seborrheic dermatitis, finding significant bacterial community differences in the middle and lower parts of hair follicles, and that the bacterial microbiome compositions in the hair follicles of healthy volunteers and patients with seborrheic dermatitis were different.

As acne occurs centered in hair follicles, it is reasonable to differentiate the microbiome within hair follicles from that from other skin locations, just as Lousada *et a.l* said, “A truly comprehensive understanding of human HF biology and pathology is not conceivable without deeper knowledge of the HF microbiome in each of the epithelial and mesenchymal HF compartments” (18). Moreover, due to the fact that not each hair follicle in acne-affected areas develops acne, therefore differentiating the microbiome of lesional follicles from non-lesional ones seems more plausible. Using more accurate sampling methods to obtain microbiome samples of certain niches specifically allows for the differentiation of lesional and non-lesional data, thus facilitating the exploration of possible patterns and connections between microbiome and acne.

The study of microecology within HF is far from sufficient. Most acne related skin microbiome studies used swabbing or stripping based sampling methods, which are unable to differentiate microbiome of skin surface from inside follicles, or lesional from non-lesional follicles.

Our data suggest that individual comedo extraction (S3) should be considered the most suitable method for acne intrafollicular microbiome studies. This method directly targets inside lesional follicles, where dysbiosis is most pronounced. While MSSSB (S2) effectively collects intrafollicular samples but does not differentiate lesional from non-lesional follicles, it is suitable for general intrafollicular microbiome studies. Swabbing (S1) is limited to surface microbiota sampling.

Our proposal and data are seemingly inconsistent to some previous research on skin microecology. For instance, Kazuhiro Ogai et al. (19) reported the species of microbes obtained by different methods are similar in normal skin. This can be explained by the fact that “species” NAMES and QUANTITIES are different concepts. A set of same species do not necessarily form identical microbial communities due to the fact that on such occasion, it is the quantity (abundance) variations characterize the microbial community, and when the variations reach a certain degree, dysbiosis is caused. Comparing the quantitive data (relative abundance) between a “healthy” and “unhealthy” microbiome comprised by similar microbial species (names) allows us to find the key microbial species that play a role. Therefore, when those skin conditions involving special ecological niches (such as hair follicles) are considered, specific sampling method targeting certain niches and more meticulous sample processing methods should be considered.

This study also reminds us that sampling and analytical methods should be carefully considered when interpreting microecological data from different studies. The impact of sampling methods on microbiome data may not only be applicable to acne, but also to numerous other skin conditions related to pilosebaceous units, such as rosacea, seborrheic dermatitis, and enlarged pores. There is a strong need to harmonize sampling and analysis methods in relevant studies in future researches. We propose and highlight the idea of accurate microbiome sampling methods with an aim to further enhance our insights into skin microecology and skin health research.

## Data Availability

The original contributions presented in the study are included in the article/**Supplementary Material**. Further inquiries can be directed to the corresponding author.
