# Supplementary material for: A noteworthy issue: microbiome data variation depending on sampling methods in skin microecology studies in acne vulgaris patients
Source: Front Immunol. 2025 Jun 9;16:1566786. doi: 10.3389/fimmu.2025.1566786 (PMC12183089; doi:10.3389/fimmu.2025.1566786)
Supplement: Supplementary file 1 [file Table1.docx]

Supplement figure 1


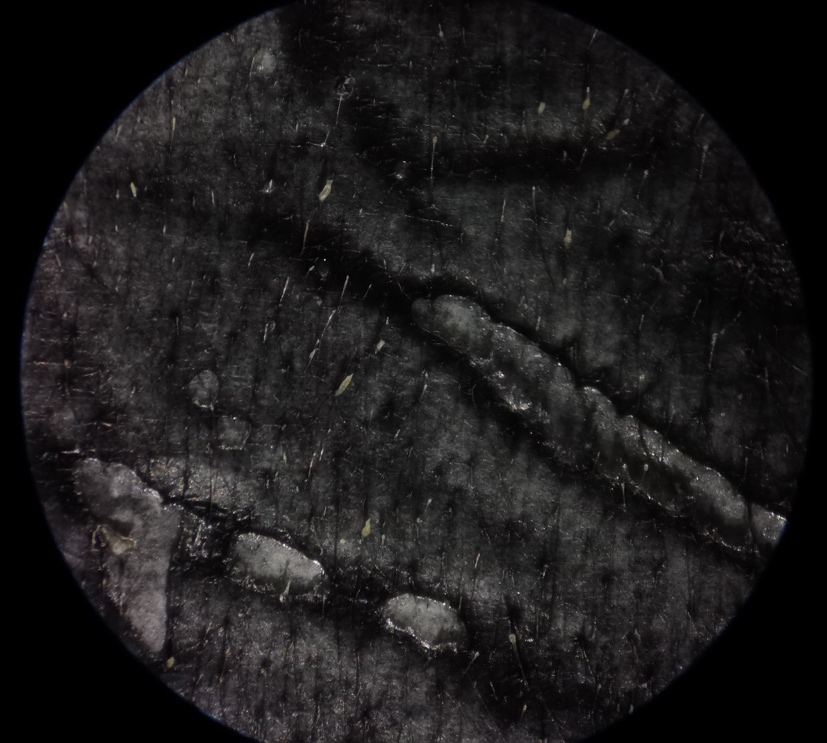


Fig. Observation of a skin follicle sample from an acne patient obtained using the MSSSB method under a stereomicroscope.

Supplement table Statistical Analysis of Optimized Bacterial and Fungal Sequencing Data

| Sample Source | Number of Samples | Number of Sequences(bp) | Base Pairs(bp) | Average Length (bp) |
| --- | --- | --- | --- | --- |
| bacterial 16S rRNA（V3-V4） | 30 | 1454362 | 605833335 | 416.56 |
| fungal 18S ITS | 30 | 1406999 | 371323734 | 263.91 |

Supplement method

Protocol of DNA amplification, purification, sequencing, and analysis

The quality of PCR products was checked by gel electrophoresis. The gel was then purified using the AxyPrep DNA Gel Extraction Kit (Axygen Biosciences, Union City, CA, USA). Subsequently, the concentration of the purified PCR products was quantified with a QuantiFlurorTM-ST flurometer (Promega).

Purified DNA was sent to Biozeron Co. Ltd. in Shanghai for constructing clone libraries and sequencing. Sequencing reads were assembled and screened. High-quality sequences were clustered with a 97% identity threshold using Usearch. After discarding singletons, each cluster was designated as an operational taxonomic units (OTUs). α-Diversity and β-Diversity were calculated.
